# Post-Retirement Paid Work and Inequalities at Older Ages

**DOI:** 10.1093/geroni/igab046.1602

**Published:** 2021-12-17

**Authors:** Benjamin Shaw, Kevin Cahill, Michael Giandrea

## Abstract

Participation in paid work frequently extends beyond pensionable age, with the Organization for Economic Cooperation and Development observing, in “Pensions at a Glance” (2017, pp. 126–7), that effective retirement ages in high-income countries exceed normal full-pension-eligibility ages by 10 months for men and two months for women. While working after pensionable age is becoming ever more common, not all workers on the cusp of retirement are able to continue in their current position or find a new job. Remarkably, little is known about the implications of unequal access to post-retirement work for social and income inequalities in later life, nor how job quality might change as people work into the years normally set aside for retirement. The four papers in this symposium address the following questions: 1) do bridge employment transitions exacerbate or mitigate income inequality later in life? 2) how does job quality (job satisfaction, physical and psychosocial working conditions) compare before and after pensionable age? 3) which processes lead to changes in working conditions in the late career? and 4) might empirical and theoretical gains be made by considering post-pensionable-age paid work as a specific career stage? The presenters use longitudinal data from the United States (the Health and Retirement Study, HRS), Sweden (Swedish Longitudinal Occupational Survey of Health, SLOSH), and Japan (Japanese Study of Aging and Retirement, JSTAR) complemented by interviews with older workers in Sweden. This symposium will provide insights into the nature and consequences of working after pensionable age in contrasting institutional settings.

